# Sildenafil and furosemide nanoparticles as a novel pharmacological treatment for acute renal failure in rats

**DOI:** 10.1007/s00210-024-03128-1

**Published:** 2024-05-15

**Authors:** Mahmoud S. Sabra, Essmat A. H. Allam, Khaled M. Ahmed Hassanein

**Affiliations:** 1 Pharmacology Department, Faculty of Veterinary Medicine, Assiut University, Assiut, 71526 Egypt; 2 Department of Pharmacology and Toxicology, Faculty of Pharmacy, Assiut University, Assiut, 71526 Egypt; 3 Pathology and Clinical Pathology Department, Faculty of Veterinary Medicine, Assiut University, Assiut, 71526 Egypt

**Keywords:** Acute renal failure, Glycerol, Sildenafil, Furosemide, NGAL, Caspase-3

## Abstract

Hospitalized patients often develop acute renal failure (ARF), which causes severe morbidity and death. This research investigates the potential renoprotective benefits of sildenafil and furosemide in glycerol-induced ARF, and measures kidney function metrics in response to nanoparticle versions of these medications. Inducing ARF is commonly done by injecting 50% glycerol intramuscularly. Rats underwent a 24-h period of dehydration and starvation before slaughter for renal function testing. We investigated urine analysis, markers of oxidative stress, histology of kidney tissue, immunohistochemistry analysis of caspase-3 and interleukin-1 beta (IL-1 β), kidney injury molecule-1 (KIM-1), and neutrophil gelatinase–associated lipocalin (NGAL), which are specific indicators of kidney tissue damage. The results of our study showed that the combination of sildenafil and furosemide, using both traditional and nanoparticle formulations, had a greater protective effect on the kidneys compared to using either drug alone. The recovery of renal tissue indicators, serum markers, and urine markers, which are indicative of organ damage, provides evidence of improvement. This was also indicated by the reduction in KIM-1 and NGAL tubular expression. The immunohistochemistry tests showed that the combination therapy, especially with the nanoforms, greatly improved the damaged cellular changes in the kidneys, as shown by higher levels of caspase-3 and IL-1β. According to the findings, a glycerol-induced rat model demonstrates that sildenafil and furosemide, either alone or in combination, in conventional or nanoparticulate forms, improve ARF dysfunction. The synergistic nanoparticulate compositions show remarkable effectiveness. This observation highlights the possible therapeutic implications for ARF treatment.

## Introduction

Acute renal failure (ARF) refers to an abrupt reduction in kidney function, which can range from minor biochemical indicators to the need for artificial renal support (Cleto-Yamane et al. 2018). Often having a complicated etiology, it is a serious complication that increases considerable morbidity and mortality, with reported death rates ranging from 11 to 63% in pediatric patients and critically ill adults (Schneider et al. 2010).

Men with erection dysfunction and pulmonary arterial hypertension receive prescriptions for phosphodiesterase type 5 (PDE5) inhibitors due to their vasodilatory effects (Newby 2022). Sildenafil, vardenafil, and tadalafil are PDE5 inhibitors; they inhibit the degradation of cyclic guanosine monophosphate (cGMP), which presumably explains their wider effects. Tritypes of nitric oxide synthase enzymes change l-arginine into nitric oxide (NO) before they work with cyclic GMP. In order to convert guanosine triphosphate to cyclic GMP, soluble guanylyl cyclase is activated by NO, a gas molecule that diffuses across the plasma membrane (Fiscus 2002; Sanders 2020). Investigations have shown that PDE5 inhibitors enhance endothelial function and shield the kidneys from damage caused by ischemia–reperfusion (Coskuner and Ozkan 2021; Morsy et al. 2014; Siregar et al. 2019). Because of their antioxidant, vasodilatory, and anti-apoptotic properties, PDE5 inhibitors show great promise as a therapy for ischemia–reperfusion damage in several organs (Abdel-Wahab et al. 2020; Hamdy et al. 2022; Thapa et al. 2021).

Loop diuretics prevent the renal tubule’s ascending limb of the loop of Henle from reabsorbing water by inhibiting Na^+^-K^+^-2Cl-co-transport. In addition, they improve the excretion of potassium, magnesium, hydrogen, and chloride in urine. The most commonly prescribed loop diuretic, furosemide, treats fluid excess in end-stage renal disease and congestive heart failure, alleviating edema and breathing difficulties (Carone et al. 2016). Loop diuretics can also help prevent intra-tubular blockage by increasing urine flow by removing debris and damaged epithelium. To some extent, ARF could benefit from this loop diuretic mechanism (Patschan et al. 2019; Schetz 2004).

Nanoparticles (NPs) are being targeted to kidney tissues by researchers because they can carry drugs directly to sick tissue, make drugs more tolerable overall, and reduce side effects in people with renal disease who need to take drugs for life (Huang et al. 2021). Because of their poor pharmacokinetic properties—such as a shorter renal retention time, lower accumulation at the illness site, and systemic side effects—medications targeted at kidney tissues are in high demand (Williams et al. 2018). One potential solution for ARF is to create drug-loaded NPs that can concentrate in the kidneys. Animal experiments are studying strategies based on NPs to find the best ways to transport molecules and increase retention in the kidney (Yu et al. 2020).

In the course of an extensive review of the current studies, we identified a significant research gap on the effects of nanoparticulate formulations of sildenafil and furosemide, on their own and when combined, on ARF that has not been previously investigated. This underscores the pioneering nature of our study.

## Materials and methods

### Experimental animals and induction of acute renal failure

The ethics committee of the Faculty of Veterinary Medicine at Assiut University gave its approval to the experimental technique (approval number: 6/2024/0169). The experiment included adult male albino rats that weighed between 150 and 250 g. Under normal laboratory circumstances, the animals were housed in the animal home of Assiut University’s Faculty of Veterinary Medicine, which included a 12-h light–dark cycle and unrestricted access to water and food. Each experimental group consisted of six animals assigned at random. The injection of 50% glycerol (8 ml/kg) causes ARF. Both hind legs receive a deep intramuscular injection to deliver the required dosage of glycerol. Before being treated with glycerol, rats were fasted for 24 h. After that, they were sacrificed to examine their renal function (Singh et al. 2011).

For the 24-h urine samples, rats were kept in metabolic cages with unrestricted access to food and drink. Prior to scarification, serum was taken from blood samples obtained at the end of the trial. We put the rats to sleep by inhaling 5% isoflurane before euthanasia. We swiftly terminated the rats’ lives by neck dislocation when they failed to react to head and limb stimulation. Rats were considered dead after 10 s of cervical dislocation if they ceased breathing and did not respond to systemic stimulation. We removed tissue samples from the kidneys, homogenized them, and then stored them at − 80 °C. Ten percent phosphate-buffered formalin was used to preserve kidney tissues for histopathological and immunohistochemical analysis (Sabra et al. 2023; Hamdy et al. 2022).

### Experimental protocol

Each of the ten groups consisted of six rats. One set of rats was given saline as a control. In group 2, known as the ARF group, glycerol causes ARF. Oral administration of 1 mg/kg of plain sildenafil dissolved in saline was provided to the third group of rats as soon as they were ARF-induced. The fourth group of rats was given regular furosemide (20 mg/kg i.m.) along with ARF as soon as it was induced. Group 5 animals were immediately administered a combination of furosemide (20 mg/kg i.m.) and sildenafil (1 mg/kg p.o.) along with ARF as soon as it was induced. Group 6 animals received sildenafil-loaded NPs in addition to being ARF-induced. Group 7 animals were administered NPs laden with furosemide and were also stimulated with ARF. Group 8 animals received a combination of sildenafil- and furosemide-loaded NPs in addition to being ARF-induced. Animals in group 9 were given carrier-based NPs of chitosan (CS), and were also induced with ARF. Animals in group 10 were given carrier-based NPs of polylactic-co-glycolic acid (PLGA) and were also induced with ARF.

### Methods for creating nanoparticles of chitosan and alginate that contain furosemide

Furosemide loading NPs were conducted in Egypt’s National Research Center. Our previous work focused on determining the best methods for preparing NPs (Hamdy et al. 2022).

### The process of creating nanoparticles of polylactic-co-glycolic acid that contain sildenafil

Sildenafil-loading NPs were developed by Egypt’s National Research Center. To generate NPs loaded with sildenafil, the solid-in-oil-in-water (s/o/w) emulsion method was employed. In order to create a PLGA solution with consistent particle size, 35 mg of PLGA was dissolved in dichloro-methane for a duration of 6 h. After mixing the PLGA solution with 3 mg of regular sildenafil and subjecting it to sonication at 55 W for 1 min, the main emulsion of solids in oil was created. Twenty milliliters of a polyvinyl alcohol solution (1% w/v) was ultrasonically treated with 55 W for 2 min to get the finished solid-in-oil-in-water emulsion. To provide room for the solvent to evaporate, the nano-sized particles whirled about in the emulsion for 3 h. Centrifugation was used for 15 min at 15,000 rpm to extract any remaining solvent from the emulsion. Prior to resuspending in deionized water and drying on a lyophilizer, the NPs were washed three times with deionized distilled water. Until they were needed again, the NPs were stored at 4 °C (Ghasemian et al. 2013).

### Transmission electron microscopy

Using a transmission electron microscope (TEM) JEM-2100 HR (Jeol, USA), the researchers at Egypt’s National Research Center examined the NPs’ size and morphology using a high-resolution transmission electron microscope at an accelerating voltage of 200 kV. Staining with a solution of 1% (w/v) sodium phosphotungstate was applied after the lyophilized drug-NP solution (1 mg/ml) was placed on copper grids coated with nitrocellulose membrane. Around 15 min after depositing the NPs, we placed the grid into the TEM to determine its size and morphology (Asadi Asadabad and Jafari Eskandari 2015).

### Measurements of zeta potential

According to Abouelhag et al. (2017), the measurements of zeta potential were made using a zeta potential analyzer (National Research Center, Egypt). Using a standard approach and disposable zeta cells, the zeta potential was measured in double-distilled water at 25 °C. The device was frequently calibrated with a − 50-mV latex standard. Yien et al. (2012) used the phase analysis light scattering approach to calculate the mean zeta potential.

### Determination of urinary albumin

A commercially available assay kit was utilized for the measurement of albumin. Bromcresol green, which produces a colorful product with albumin, is utilized in this method. We evaluate the color intensity at 620 nm to determine the amount of albumin in the sample (Zhao et al. 2020).

### Determination of urinary glucose

A glucose test kit that is available for purchase was used for this purpose. Glucose was measured after enzymatic oxidation by glucose oxidase. Under the catalytic action of peroxidase, the produced hydrogen peroxide reacts with phenol and 4-aminoantipyrine to produce the violet quinonimine color. The color intensity at 560 nm directly correlates with glucose concentration (Saleh et al. 2012).

### Determination of urinary ketone bodies

The presence of ketones was assessed with the use of an easily available test kit. The acetoacetic acid and 3-hydroxybutyric acid concentrations are directly correlated with the nicotinamide adenine dinucleotide absorbance at 340 nm, which is the basis of the ketone body test, which depends on reactions that are facilitated by 3-hydroxybutyrate dehydrogenase. You can tell how much acetoacetic acid and 3-hydroxybutyric acid are in a sample by looking at the color intensity at 340 nm (Nuwayhid et al. 1988).

#### Assessment of renal function

Using commercially available test kits (Schiffgraben, Hannover, Germany) according to the manufacturer’s instructions, evaluations of renal function were conducted by measuring blood urea nitrogen and serum creatinine. We measure the aforementioned characteristics using spectrophotometry.

#### Markers of oxidative stress

Nitric oxide levels in kidney tissue homogenates were evaluated, and malondialdehyde (MDA) levels were measured spectrophotometrically with a commercially available kit from Schiffgraben in Hannover, Germany.

#### Specific biomarkers for renal failure

The proximal tubule apical membrane of injured rats produces the type-1 transmembrane protein known as kidney injury molecule-1. The KIM-1 level was assessed by means of an ELISA kit that was manufactured and sold by Sunlong Biotechnology in Shanghai, Hang-zhou, Zhejiang, China (Gohda et al. 2020).

When the proximal convoluted tubule of a rat’s kidney is damaged, it makes more neutrophil gelatinase–associated lipocalin (NGAL), a protein in the lipocalin superfamily. Sunlong Biotechnology of Shangyi, Hangzhou, Zhejiang, China, supplied the ELISA kit that was used to test NGAL (Sabra et al. 2023).

#### Histopathology and immunohistochemistry

We used a 10% neutral buffered formalin solution to fix the kidney tissue samples. Next, we dehydrate the samples using progressively stronger alcohols, clear them with xylene, and finally embed them in paraffin. Hematoxylin and eosin (H&E)–stained 5-micron-thick tissue sections (Perry et al. 2016).

Interleukin-1 beta (IL-1β) and caspase-3 were examined using immunohistochemical labeling. After deparaffinization in xylene, 4-µm tissue sections were rehydrated. After 10 min in 3% H_2_O_2_ to eliminate endogenous peroxidase activity, the sections were washed twice with PBS for three washes. Following the typical manufacturing technique, the sections were incubated with normal goat serum at 37 °C (Vector Laboratories, Burlingame, CA). Next, they were treated for 1 h at room temperature with primary rabbit anti-caspase-3 antibody (E-AB-6602, Elabscience Biotechnology, USA) at 1/200 dilution and rabbit anti-IL-1 β antibody (1/100) (E-AB-66749, Elabscience Biotechnology inc., USA). Next, polyperoxidase-anti-mouse/rabbit IgG was added for 20 min. The antigen–antibody combination was identified using a streptavidin–biotin-peroxidase kit after Mayer’s hematoxylin counterstaining. Each experiment contained positive and negative controls. We counted immunopositive cells in five microscopic regions on each slide. After calculating the numbers, we calculated the mean ± SE for each group.

#### Statistical analysis

Data for each evaluated parameter were tested for the normality of distributions (Shapiro–Wilk test, *p* > 0.05). Statistical significance was assessed by a one-way ANOVA for repeated measures, followed by Bonferroni’s multiple comparison test. The comparisons between the two groups were performed using the Student’s *t*-test.* p* ≤ 0.05 was considered statistically significant. Data are presented as means ± SE. Graph Pad Prism® software (version 8) was used to carry out these statistical tests.

## Results

### Description of nanoparticles’ physical and chemical properties

#### Characteristics of furosemide-loaded nanoparticles

**Fig. 1:**
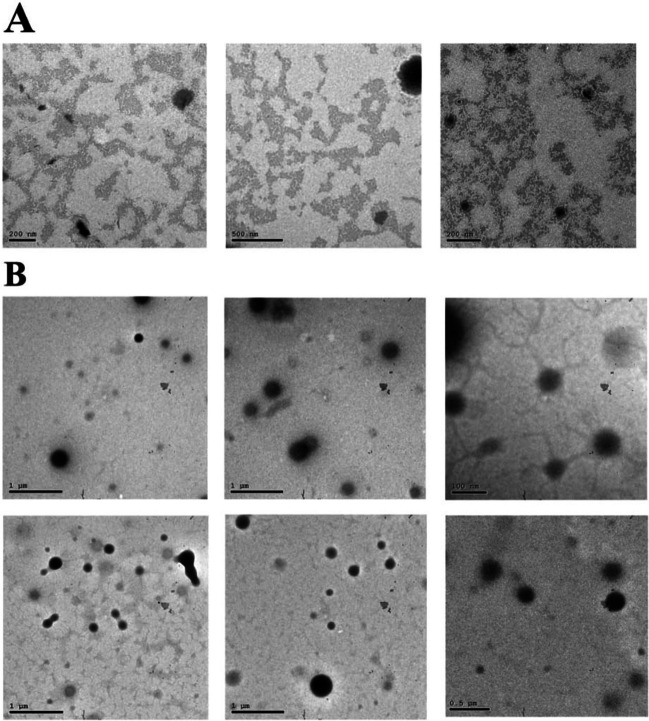
Photomicrographs taken using transmission electron microscopy (TEM) showing, at varying magnifications, the spheroidal shapes of furosemide nanoparticles (**A**) and the typical spherical shape of sildenafil nanoparticles (**B**)

**Fig. 2:**
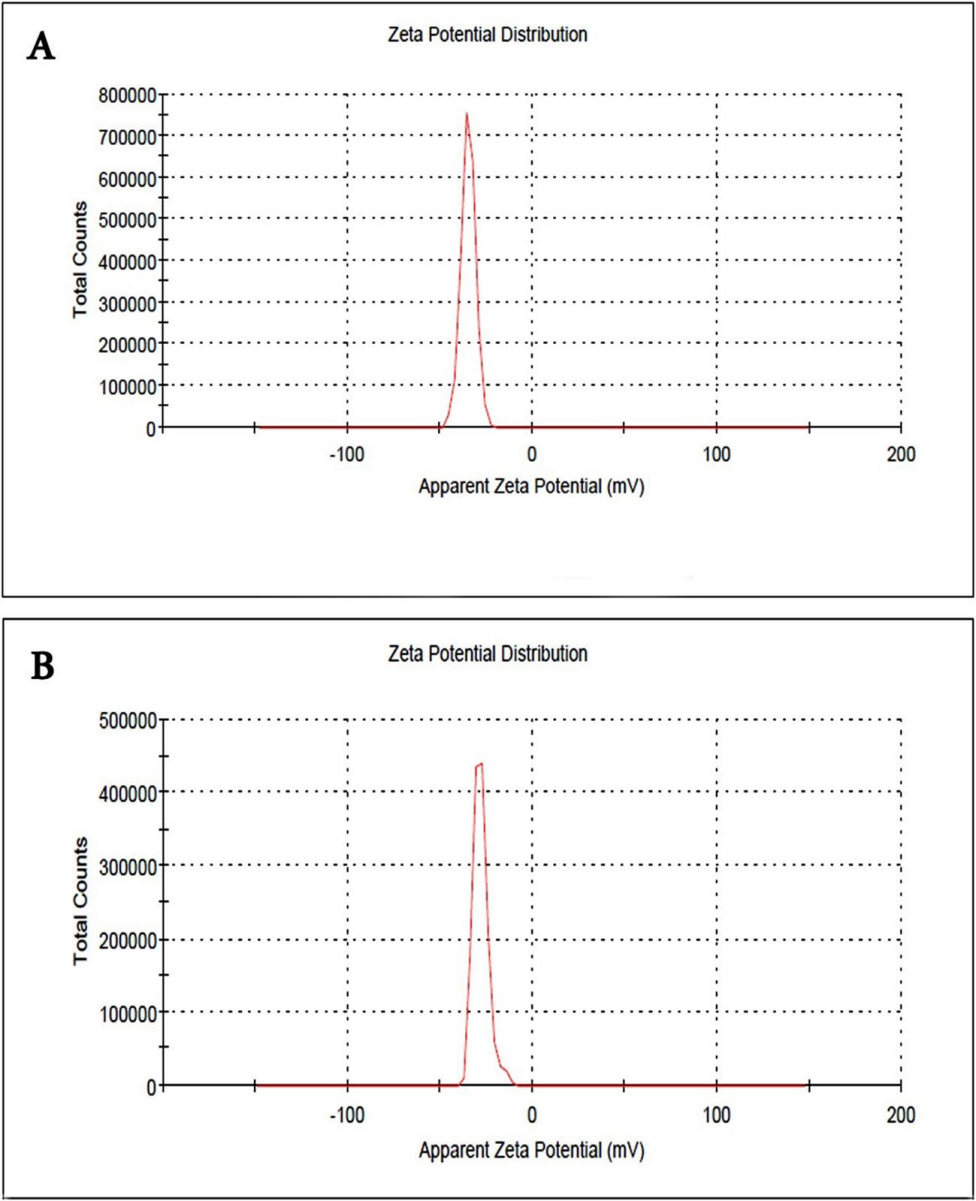
The distribution of zeta potential in chitosan/alginate nanoparticles (**A**) and the loading of furosemide into them (**B**)

Consistent with our earlier work, we prepared NPs loaded with furosemide (Hamdy et al. 2022). A TEM picture (Fig. 1A) shows that the furosemide-loaded NPs in suspension are spherical and less than 55 nm across. The National Research Center in Egypt used a Zeta Potential Analyzer to measure the charge density of chitosan/alginate nanoparticles, which was − 34.7 Mv (Fig. 2A). This charge density dropped to − 28 Mv (Fig. 2B) when furosemide was added to the chitosan/alginate. Nanocomposite values of furosemide, chitosan, and alginate cause particles with low zeta potential to flocculate because there is no force that separates them (Mekhamer 2010).

#### Characteristics of sildenafil-loaded nanoparticles

**Fig. 3:**
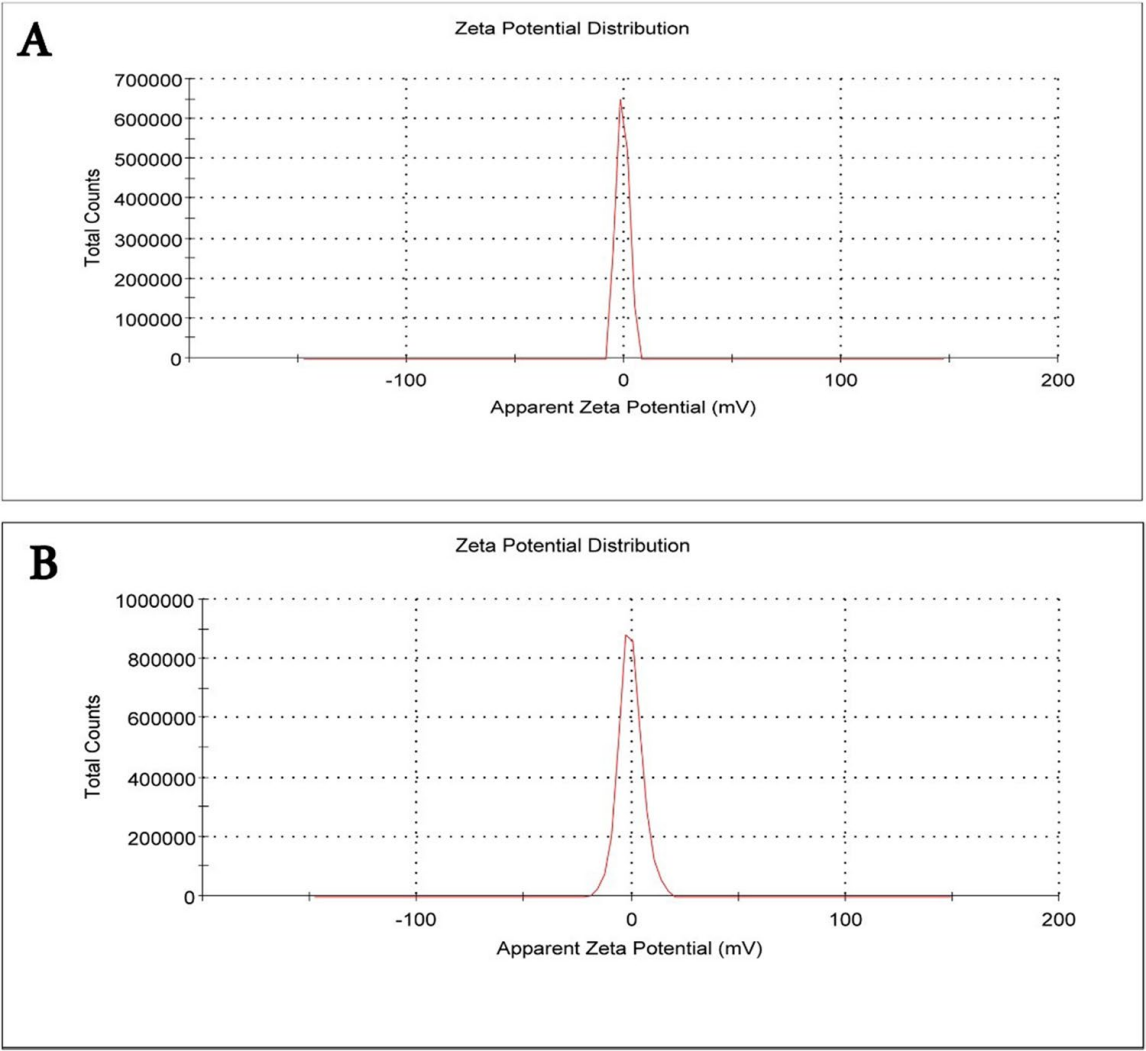
Polylactic acid-co-glycolic acid nanoparticles zeta potential distribution (**A**) and sildenafil loading in these nanoparticles (**B**)

Ghasemian et al. (2013) describe how to prepare sildenafil-loaded NPs. The current work used the nanomaterial PLGA. The NPs that contain sildenafil are 200 ± 50 nm in diameter and have a regular spherical shape and suspension form, as shown in TEM images (Fig. 1B). Using a Zeta Potential Analyzer (National Research Center, Egypt), we discovered the charge density of PLGA NPs to be − 0.778 Mv (Fig. 3A), which reduced to − 0.453 Mv (Fig. 3B) upon loading sildenafil into PLGA. Particles with low zeta potential that appeared with sildenafil-PLGA nanocomposite will flocculate because there is no force keeping them apart (Mekhamer 2010).

### The impact of sildenafil, furosemide, and their nanoparticle formulations on urinary albumin, glucose levels, and ketone bodies in rats induced with ARF

**Fig. 4:**
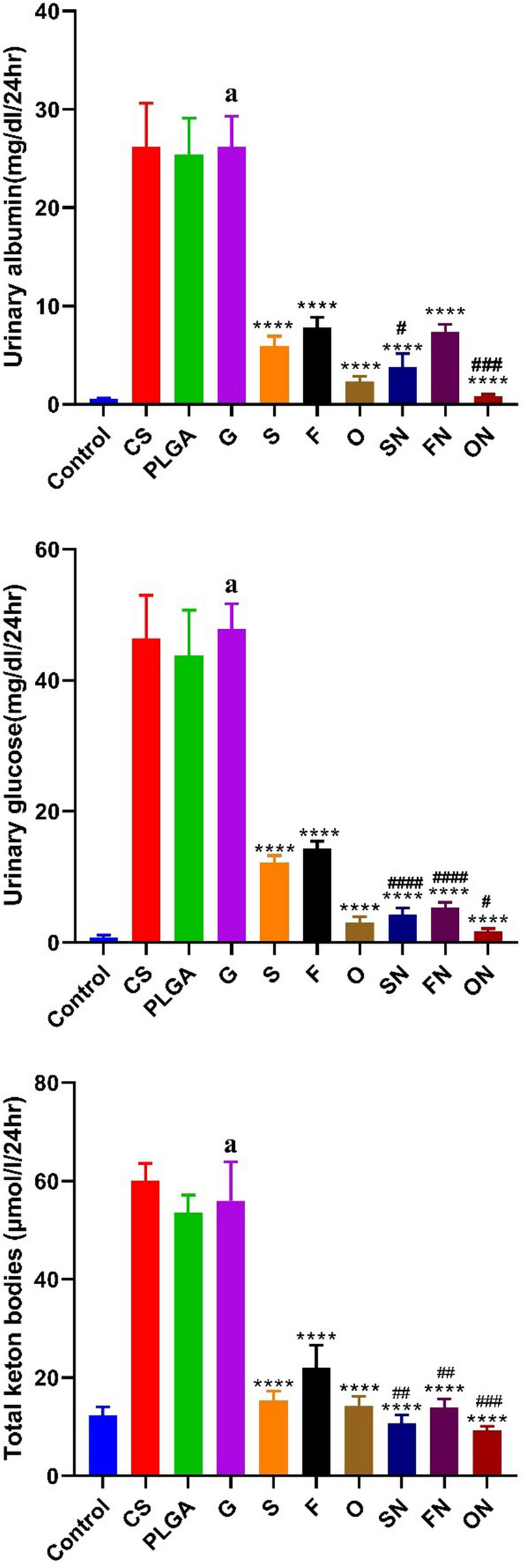
The effects of conventional, nanoparticle (N), and combination (O) forms of furosemide (F) and sildenafil (S) on albumin, glucose, and total ketone body levels in rat urine during glycerol-induced acute renal failure. Data are the means ± SEM (*n* = 6). ^a^*p* < 0.0001 as compared with the control group. ****p < 0.0001 as compared to the glycerol-treated group. ^#^*p* < 0.05, ^##^*p* < 0.01, ^###^*p* < 0.001, and ^####^*p* < 0.0001 when contrasted with the related nanoparticle category

An increase (*p* < 0.0001) in urine albumin (df = 10, *F* = 10.132), glucose level (df = 10, *F* = 97.57), and ketone bodies (df = 10, *F* = 21.45) was seen in rats treated with glycerol-induced ARF (+ ve control) when contrasted with the negative control group. When contrasted with rats given AFR, animals given chitosan or PLGA showed no discernible changes. In contrast to rats induced with ARF, animals treated with conventional and NPs sildenafil, furosemide, or a combination of the two exhibited a substantial reduction (*p* < 0.0001) in urine albumin (*F*_(6,35)_ = 4.46), glucose level (*F*_(6,35)_ = 2.016), and ketone bodies (*F*_(6,35)_ = 5.112). Figure 4 shows that, compared to rats treated with their respective conventional drugs, animals given sildenafil NPs, furosemide NPs, or a combination of the two had a substantial reduction (*p* < 0.05) in the examined parameters.

### The impact of sildenafil, furosemide, and their nanoparticle formulations on blood creatinine and urea levels in rats induced with ARF.

**Fig. 5:**
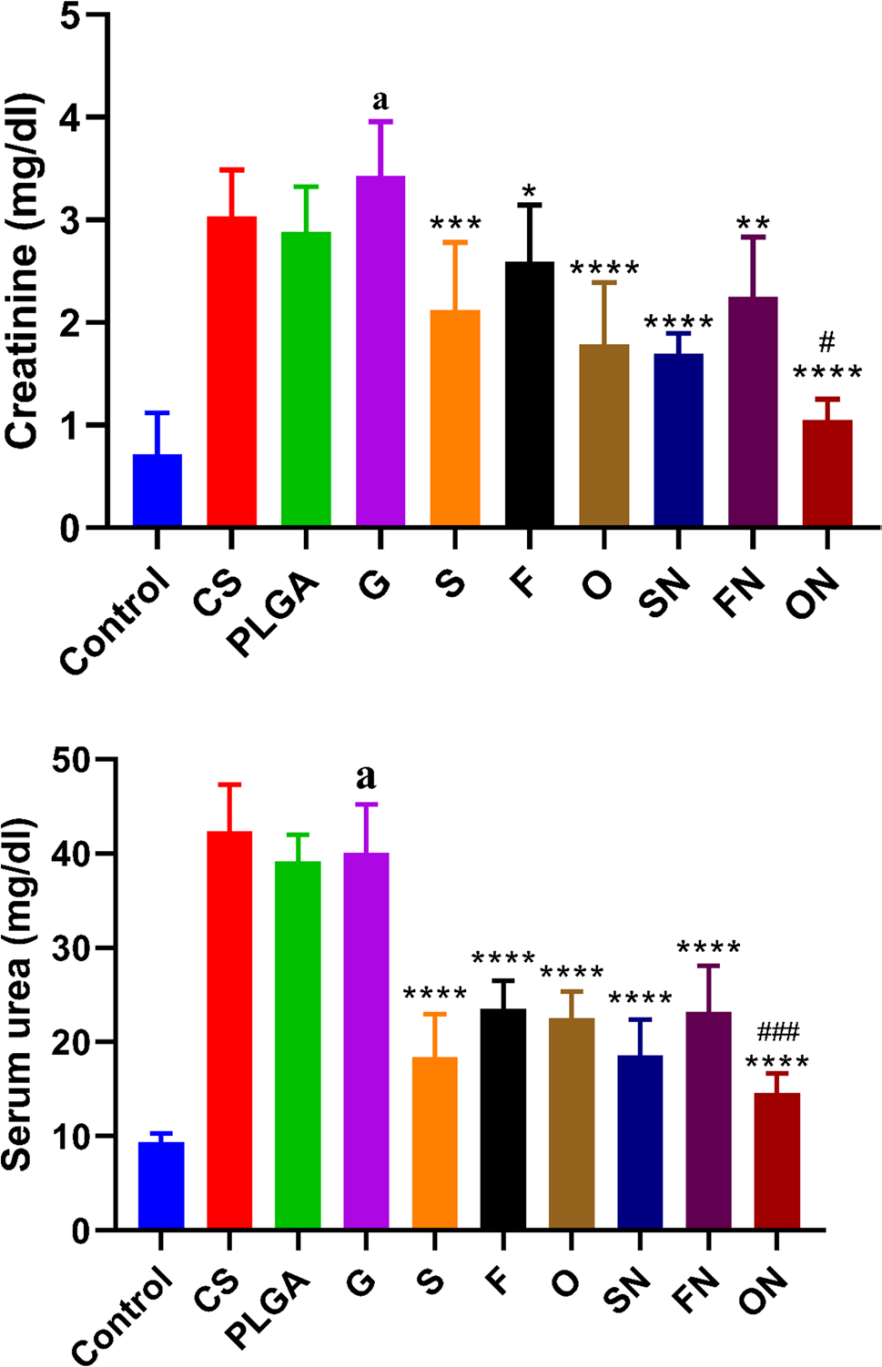
The effects of conventional, nanoparticle (N), and combination (O) forms of furosemide (F) and sildenafil (S) on blood creatinine and urea levels in rats during glycerol-induced acute renal failure. Data are the means ± SEM (*n* = 6). ^a^*p* < 0.0001 as compared with the control group. *p < 0.05, **p < 0.01, ***p < 0.001, and ****p < 0.0001 as compared to the glycerol-treated group. ^#^*p* < 0.05 and ^###^*p* < 0.001 when contrasted with the related nanoparticle category

When compared to the negative control group, rats that were given ARF had a significantly higher level of serum creatinine (df = 10, *F* = 1.770) and blood urea nitrogen (df = 10, *F* = 31.86) (*p* < 0.0001). Chitosan and PLGA-treated rats did not differ significantly from ARF-induced rats, though. Compared to rats induced with ARF, animals given conventional or NPs sildenafil, furosemide, or a combination of the two exhibited significantly lower levels of blood creatinine (*F*_(6,35)_ = 0.9557) and urea (*F*_(6,35)_ = 1.096) (*p* < 0.05). Treatment of ARF-induced rats with NP forms, especially the furosemide-sildenafil combination, resulted in a more pronounced decrease in creatinine and urea (Fig. 5).

### The impact of sildenafil, furosemide, and their nanoparticle formulations on tissue malondialdehyde and nitrite levels in rats induced with ARF

**Fig. 6:**
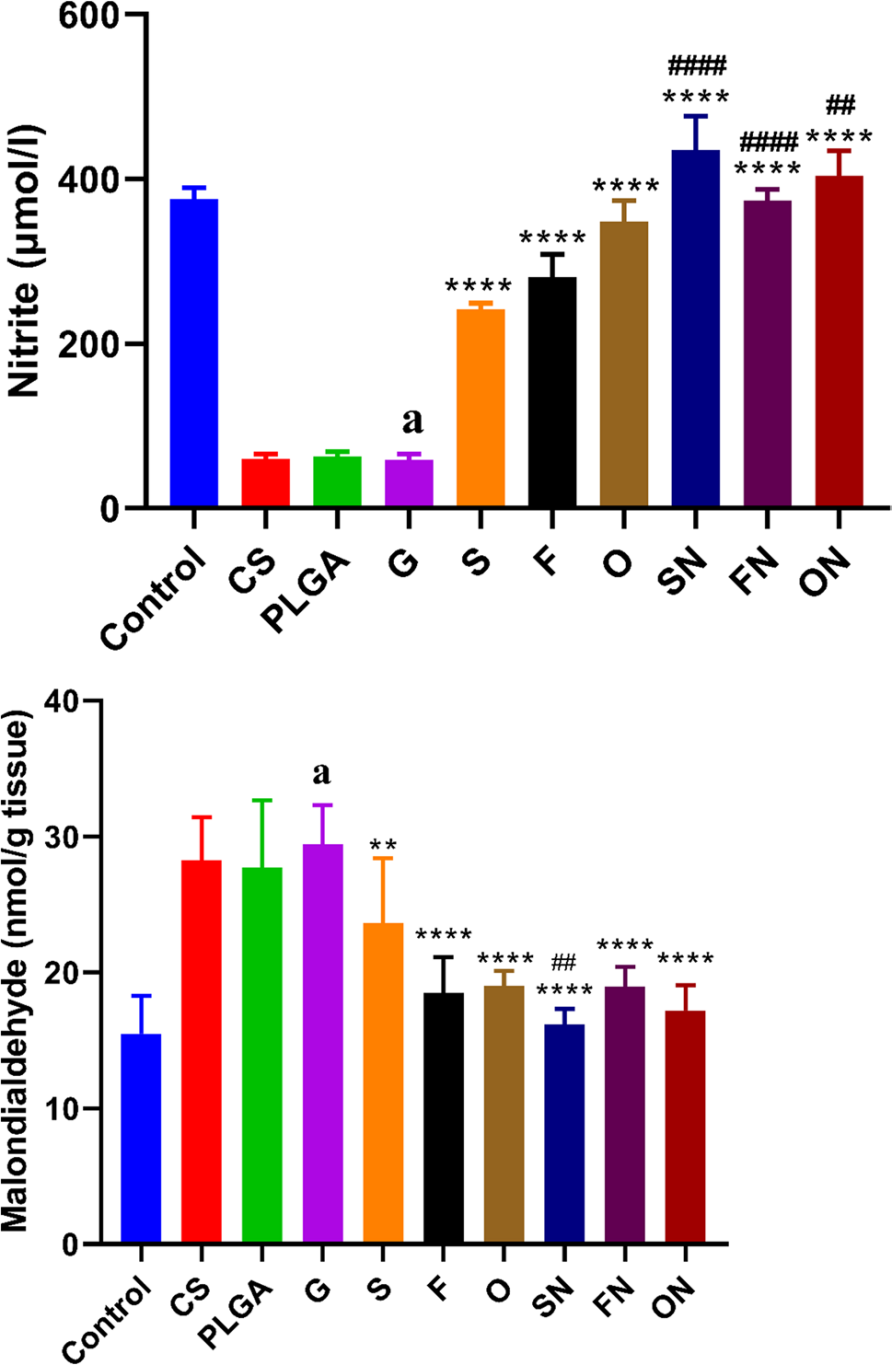
The effects of conventional, nanoparticle (N), and combination (O) forms of furosemide (F) and sildenafil (S) on tissue malondialdehyde and nitrite levels in rats during glycerol-induced acute renal failure. Data are the means ± SEM (*n* = 6). ^a^*p* < 0.0001 as compared with the control group. **p < 0.01, and ****p < 0.0001 as compared to the glycerol-treated group. ^##^*p* < 0.01 and ^####^*p* < 0.0001 when contrasted with the related nanoparticle category

Figure 6 shows that compared to the negative control group, rats that were given ARF had significantly higher levels of tissue malondialdehyde (df = 10, *F* = 1.101) (*p* < 0.0001) and much lower levels of nitrite (df = 10, *F* = 3.338) (*p* < 0.0001). Chitosan- and PLGA-treated rats did not differ significantly from ARF-induced rats, though. The tissue malondialdehyde levels were significantly decreased (*p* < 0.001) while the levels of nitrite were significantly increased (*p* < 0.0001), in comparison to the ARF-positive control group. The tissue malondialdehyde levels (*F*_(6,35)_ = 1.909) were significantly decreased (*p* < 0.001), while the levels of nitrite (*F*_(6,35)_ = 3.457) were significantly increased (*p* < 0.0001) in comparison to the ARF-positive control group when using conventional and NPs forms of sildenafil, furosemide, and their combinations. There was a notable rise in tissue nitrite (*p* < 0.01) in all nanogroups and a considerable reduction (*p* < 0.01) in tissue malondialdehyde, particularly in rats treated with sildenafil NPs for ARF when contrasted with animals given the same conventional medications.

### The impact of sildenafil, furosemide, and their nanoparticle formulations on tissue kidney injury molecule-1 and neutrophil gelatinase–associated lipocalin levels in rats induced with ARF

**Fig. 7:**
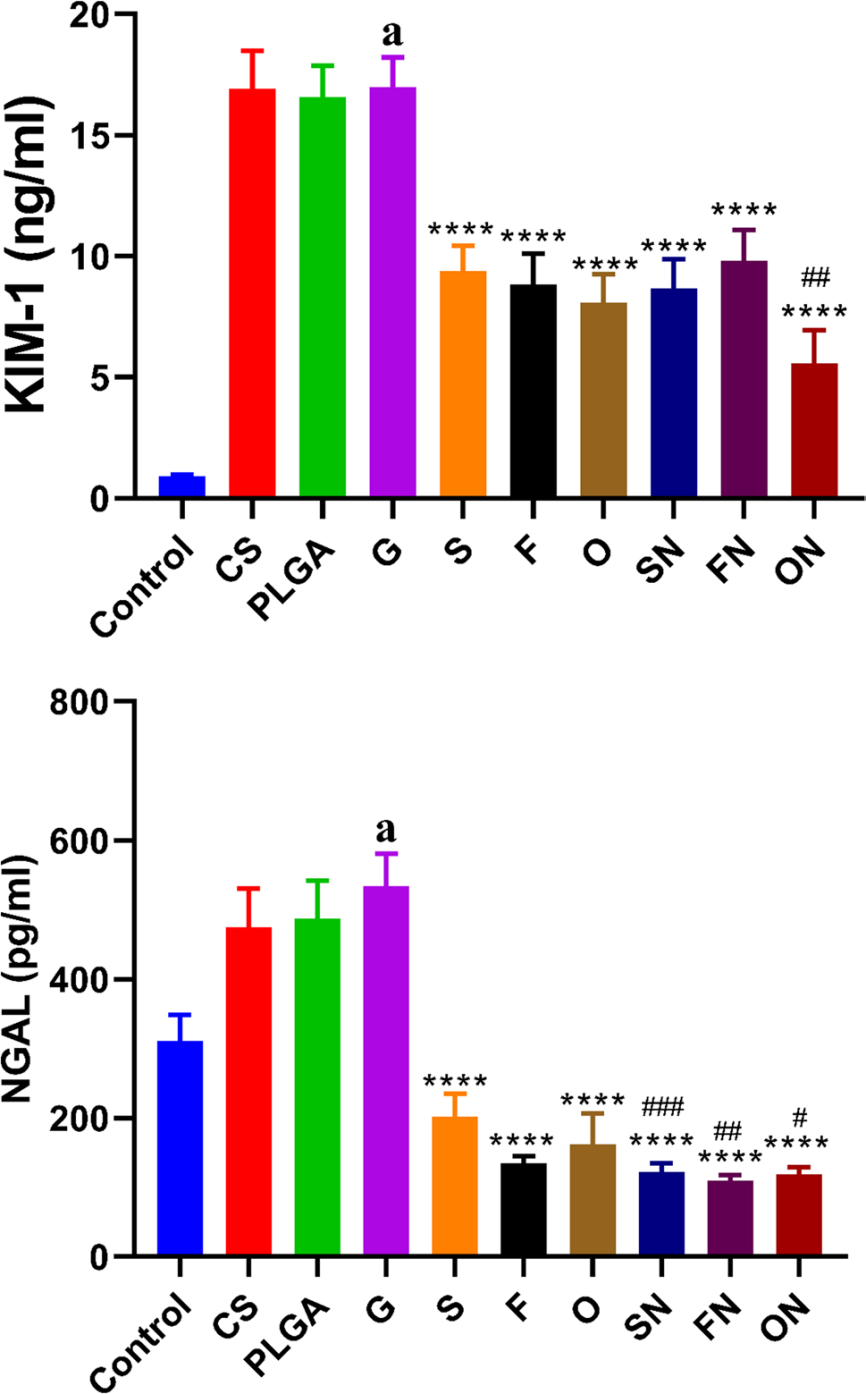
The effects of conventional, nanoparticle (N), and combination (O) forms of furosemide (F) and sildenafil (S) on tissue kidney injury molecule-1 and neutrophil gelatinase–associated lipocalin levels in rats during glycerol-induced acute renal failure. Data are the means ± SEM (*n* = 6). ^a^*p* < 0.0001 as compared with the control group. ****p < 0.0001 as compared to the glycerol-treated group. ^#^*p* < 0.05, ^##^*p* < 0.01, and ^###^*p* < 0.001 when contrasted with the related nanoparticle category

When matched to the negative control group, rats administered with glycerol exhibited significantly higher levels of tissue KIM-1 (df = 10, *F* = 20.289) (*p* < 0.0001) and NGAL (df = 10, *F* = 1.551) (*p* < 0.0001). Nonetheless, when contrasted with the ARF-positive control group, rats treated with chitosan or PLGA showed no discernible changes. In comparison to the ARF-induced group, the conventional and NP versions of sildenafil, furosemide, and their combinations showed a substantial reduction (*p* < 0.0001) in tissue KIM-1 (*F*_(6,35)_ = 0.06772) and NGAL (*F*_(6,35)_ = 3.710). It should be mentioned that, compared to rats treated with their respective conventional drugs, animals given sildenafil NPs or furosemide NPs and their combinations exhibited a notable reduction (*p* < 0.01) in tissue KIM-1 and NGAL, as seen in Fig. 7.

### Histopathological changes in glycerol-induced acute renal failure

**Fig. 8:**
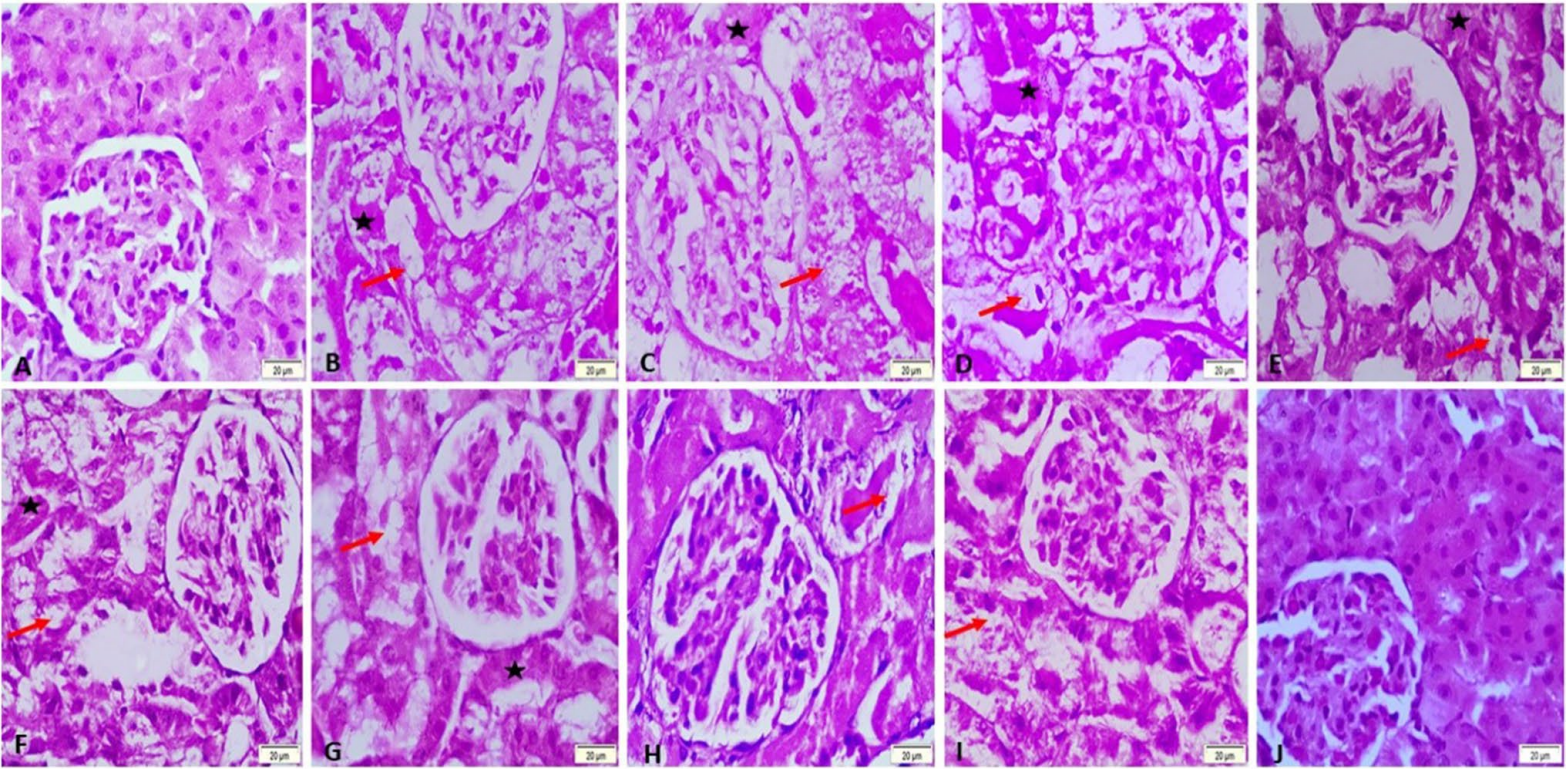
Representative micrograph of the kidney of the studied group stained by HE. **A** Control negative group showing normal histology of renal tubules and glomeruli. **B**, **C**, **D** PLGA-, chitosan-, and glycerol-treated groups, respectively, showing severe vacuolar degeneration (arrows) and coagulative necrosis of the renal tubules (stars). **E**, **F**, **G** Sildenafil, furosemide, and their combinations, respectively, show moderate vacuolar degeneration (arrows) and coagulative necrosis of the renal tubules (stars). **H**, **I**, **J** Sildenafil nanoparticle– and furosemide nanoparticle–treated groups, respectively, showing mild vacuolar degeneration (arrows). **J** Sildenafil-furosemide nanoparticle combination group showing normal appearance of the renal tubules and glomeruli

For each group that was part of the study, the histological findings of kidney lesions are documented in Table 1. The control group’s kidney sections showed normal histology of the glomerulus and renal tubular epithelium. The renal tubular epithelium showed a lot of vacuolar degeneration, coagulative necrosis, and pyknotic or karolysis nuclei in the groups of rats that were given ARF glycerol. These groups were also given PLGA and chitosan. In the groups that were given standard forms, the renal tubules showed signs of coagulative necrosis and moderate vacuolar degeneration. Mild vacuolar degeneration was observed in the group of rats treated with sildenafil and furosemide NPs, but normal appearance of the renal tubules and glomeruli was observed in the group treated with NP drug combinations in glycerol-induced ARF (Fig. 8).

**Table 1:** Summary of the lesion score of the studied groups

Groups	C	CS	PLGA	G	S	F	O	SN	FN	ON
Lesions										
-Vacuolar degeneration of the renal tubular epithelium	−	+ + +	+ + +	+ + +	+ +	+ +	+ +	+	+ +	+
-Coagulative necrosis	−	+ + +	+ + +	+ + +	+ +	+ +	+	+	+	−

No lesions, + lesions present in 1–2 sections, +  + lesions present in 3–4sections, +  +  + lesions present in 5–6 sections

*C* control group, *CS* chitosan-treated group, *PLGA* poly lactic-co-glycolic acid–treated group, *G* glycerol-induced acute renal failure group, *S* sildenafil-treated group, *F* furosemide-treated group, *O* drug combination–treated group, *SN* sildenafil nanoparticle–treated group, *FN* furosemide nanoparticle–treated group, *ON* drug nanoparticle combination–treated group

### Immunohistochemistry changes in acute renal failure

**Fig. 9:**
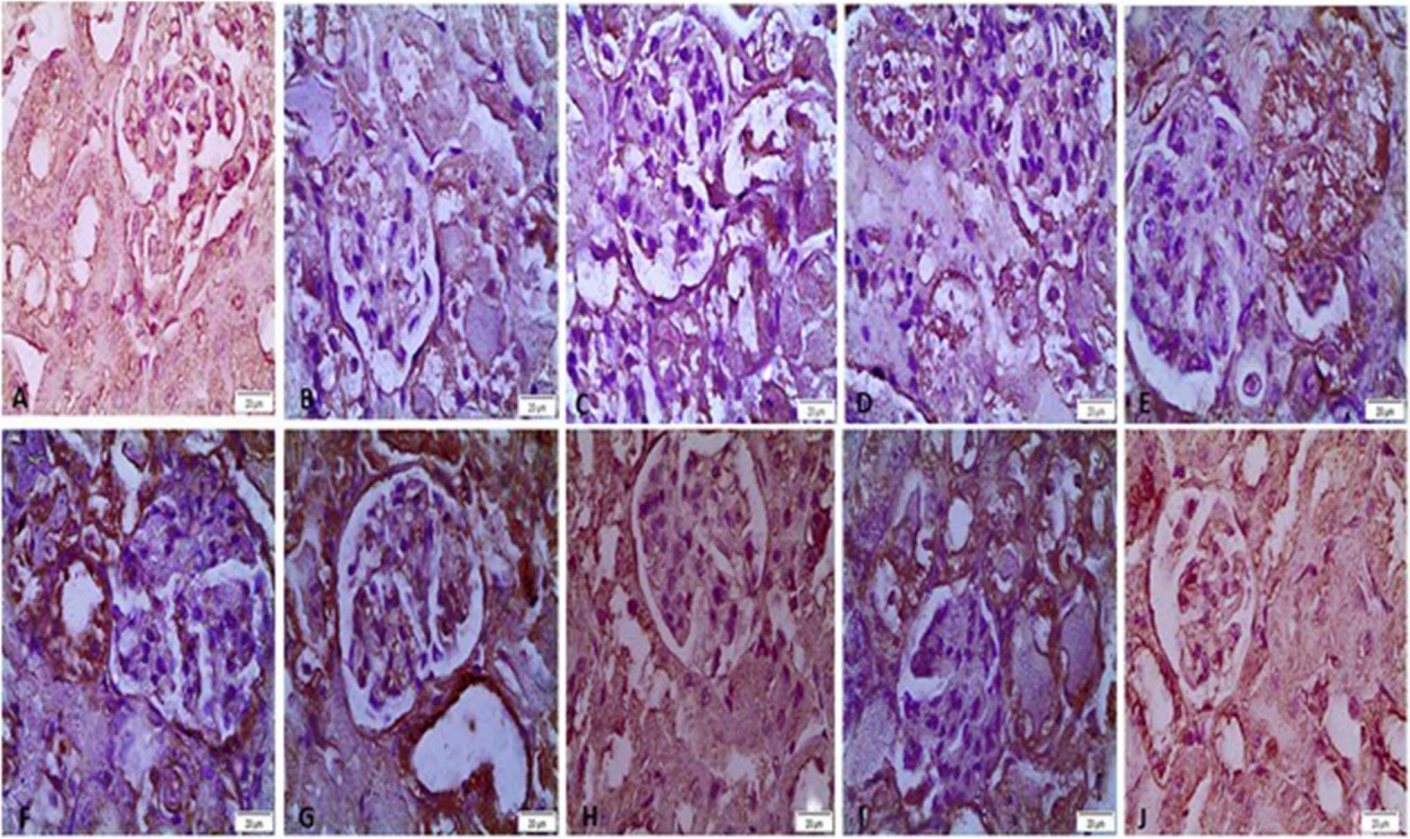
Immunohistochemical staining of caspase-3 in rat kidney. **A** The control negative group showed mild expression of caspase-3. **B**, **C**, **D** Poly lactic-co-glycolic acid–, chitosan-, and glycerol-treated groups, respectively, showed a significant increase in caspase-3 immunoreactivity in the cytoplasm of renal tubules, which appear brown. **E**, **F**, **G** Sildenafil-, furosemide-, and drug combination–treated groups, respectively, showing significant reduction in caspase-3 immunostaining. **H**, **I**, **J** Sildenafil nanoparticle–, furosemide nanoparticle–, and drug nanoparticle combination–treated groups, respectively, showed mild expression of caspase-3 immunostaining

**Fig. 10:**
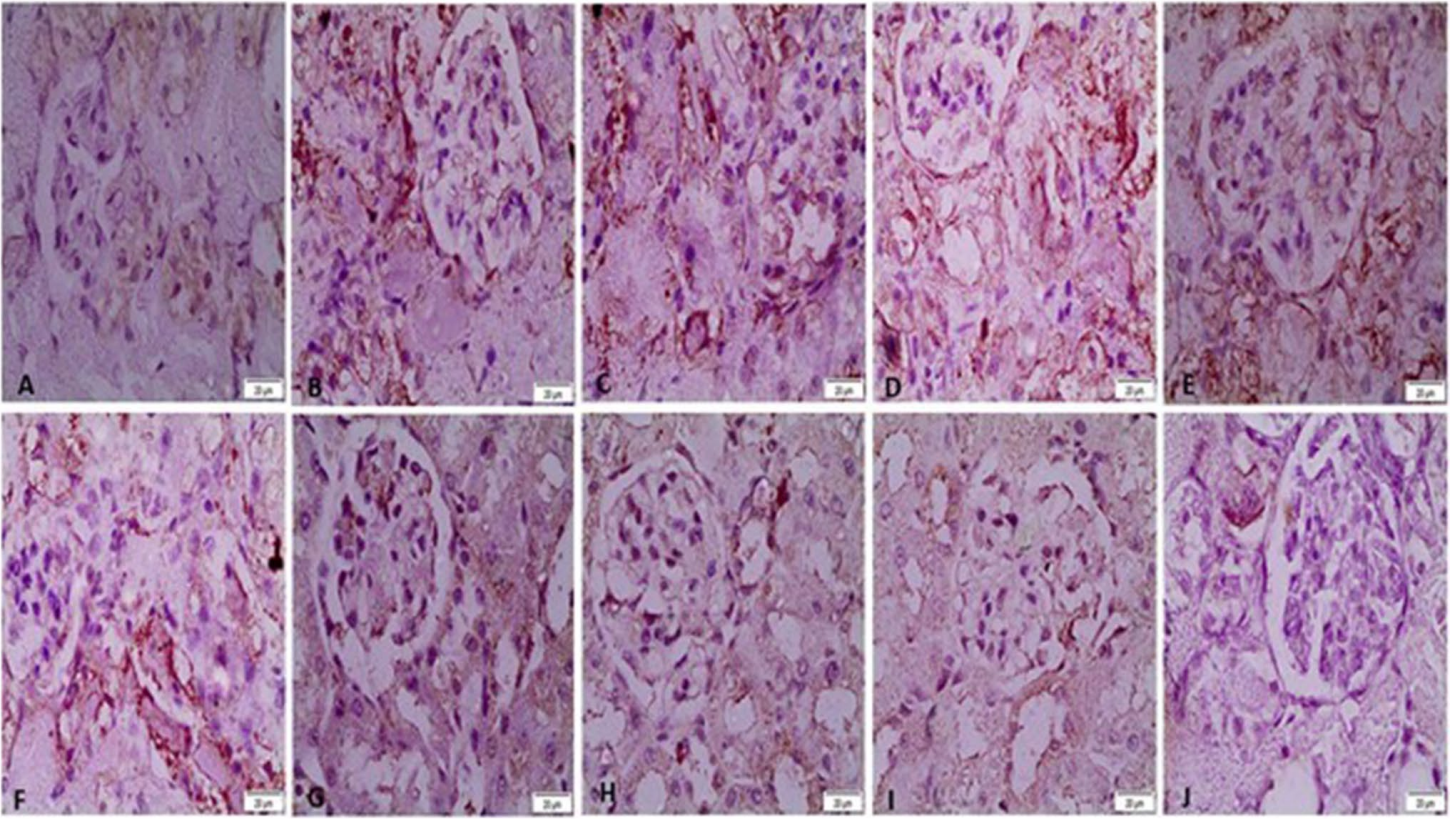
Immunohistochemical staining of IL-1β in rat kidneys. **A** The control negative group showed mild expression of interleukin-1 beta (IL-1β). **B**, **C**, **D** Poly lactic-co-glycolic acid–, chitosan-, and glycerol-treated groups, respectively, showed a significant increase in IL-1β immunoreactivity in the cytoplasm of renal tubules, which appear brown in color. **E**, **F**, **G** Sildenafil-, furosemide-, and drug combination–treated groups, respectively, showed significant reduction in IL-1β immunostaining. **H**, **I**, **J** Sildenafil nanoparticle–, furosemide nanoparticle–, and drug nanoparticle combination–treated groups, respectively, showed significant reduction in IL-1β immunostaining

Immunohistochemical staining of caspase-3 and IL-1β in rat kidneys. There was a significant increase in both caspase-3 and IL-1β immunoreactivity in the G2,3,4-treated group. Moderate expression was observed in G5, 6, and 7, and mild expression in G8, 9, and 10 (Figs. 9, 10, and Table 2).

**Table 2:** Summary of immunohistochemistry expression of caspase-3 and IL-1β in the kidney of rats in the studied groups

Groups		C	CS	PLGA	G	S	F	O	SN	FN	ON
Caspase-3		2.333 ± 0.55^a^	33.1 ± 2.9^A^	33.17 ± 2.5^A^	37.17 ± 3.4^A^	11.33 ± 1.4^B^	9.333 ± 1.1^B^	8.167 ± 1.07^B^	6.500 ± 0.9^B#^	6.167 ± 0.47^B#^	3.000 ± 0.44^B#^
IL-1β	1.833 ± 0.30^a^		14.3 ± 2.27^A^	15.7 ± 2.06^A^	21.17 ± 2.91^A^	15.00 ± 2.03^B^	14.00 ± 0.96^B^	10.83 ± 1.2^B^	6.833 ± 0.47^B#^	7.333 ± 0.88^B#^	2.333 ± 0.71^B#^

Means ± SE with different superscripts in the same row differ significantly (*p* < 0.05). Small superscript letter in comparison to control (C) group (Student’s *t*-test), large superscript letter in comparison to glycerol (G)-induced acute renal failure (one-way ANOVA, Dunnett’s post hoc test)

*CS* chitosan-treated group, *PLGA* poly lactic-co-glycolic acid–treated group, *S* sildenafil-treated group, *F* furosemide-treated group, *O* drug combination–treated group, *SN* sildenafil NP–treated group, *FN* furosemide NP–treated group, *ON* drug NP combination–treated group

^#^Student’s *t*-test to compare conventional drug groups with their corresponding nanoparticle (NP) groups (*p* < 0.05)

## Discussion

Acute kidney injury (AKI), previously known as ARF, is a clinical syndrome characterized by a rapid decline in the glomerular filtration rate (GFR). This decline leads to an accumulation of waste products and uremic toxins, impairing renal function. Despite advancements in medical management, AKI remains linked to substantial morbidity and mortality. In low- to middle-income nations, the predominant etiologies of AKI are infections and hypovolemic shock. In contrast, sepsis, drug-related effects, or invasive medical interventions frequently cause AKI in hospitalized elderly individuals in affluent countries (Kellum et al. 2021).

The NPs utilized in this research exhibit physicochemical properties, such as size, morphology, and charge density, consistent with prior studies. Their diminutive dimensions and spherical configuration augment their capability for efficient drug delivery and precision-targeted therapeutic applications. In this investigation, a glycerol injection effectively induced ARF in rats. Elevated levels of urine albumin, glucose, and ketone bodies, along with increased serum creatinine and blood urea nitrogen values in contrast to the control group, demonstrated this effect. These findings are consistent with previous studies that have highlighted glycerol’s ability to trigger rhabdomyolysis and myoglobinuric acute tubular necrosis in rats (Adedapo et al. 2020; Laham 2018; Li et al. 2019).

As previously reported (AlBasher et al. 2020), rats with glycerol-induced ARF had higher levels of malondialdehyde and lower levels of nitrite in their kidneys compared to the control group. This was a sign of oxidative stress. Furthermore, these glycerol-administered rats showed elevated concentrations of KIM-1 and NGAL. Siddiqui et al. (2019) found higher levels of KIM-1 in mice exposed to glycerol, and Sharawy et al. (2018) observed a significant rise in NGAL levels in their glycerol-treated group, indicating kidney failure.

It is interesting that the chitosan and PLGA carrier nanoparticles did not change the kidney parameters significantly when compared to the ARF-induced group. This implies that they neither adversely affected baseline renal function nor exacerbated injury mechanisms, underscoring their potential safety for drug delivery systems.

In this study, we looked at the effects of furosemide and sildenafil, both by themselves and when combined, as well as the effects of their nanoparticle forms, using a rat model of ARF caused by glycerol. Our results demonstrated that the administration of these therapeutic agents notably diminished levels of urinary albumin, glucose, and total ketones. This suggests their potential efficacy in mitigating ARF initiation within the glycerol-induced paradigm.

Surprisingly, the nanoparticle forms of sildenafil, furosemide, and their combined regimen greatly decreased the levels of albumin, glucose, and ketone bodies in the urine compared to those who were treated with the regular drug forms. This decrease may be indicative of an enhancement in glomerular filtration and renal tubular functionality, potentially driven by the diuretic and vasodilatory properties of the medications.

These results are similar to those from Hamdy et al. (2022), who found that rats given furosemide had lower levels of albumin, glucose, and ketone bodies in their urine compared to rats in the adenine-induced renal injury group. The augmented therapeutic outcomes discerned with nanoparticulation, as opposed to their traditional forms, might be ascribed to superior pharmacokinetic profiles, coupled with the heightened target specificity and bioavailability inherent to nanoformulated drugs.

Creatinine and blood urea nitrogen serve as pivotal markers for renal impairment. The GFR intrinsically linked to creatinine concentrations (van Veldhuisen et al. 2016). A decline in renal function can impede creatinine filtration, culminating in elevated serum creatinine levels. Notably, when creatinine concentrations surpass twice the standard value, it typically signifies a 50% reduction in GFR (Palmer et al. 2018). Either intensified protein breakdown or the conversion of ammonia to urea, stemming from the augmented activity of enzymes pivotal to urea synthesis, can cause a surge in blood urea nitrogen concentrations (Weiner et al. 2015).

In our study, when the medications we looked at were given to rats with glycerol-induced ARF, they significantly lowered the levels of blood urea nitrogen and serum creatinine. This underscores the reliability of these metrics as indicators of therapeutic amelioration in ARF. Nanoparticle mixtures of sildenafil, furosemide, and their combined treatment showed a significant decrease in renal injury markers compared to animals that were treated with regular drugs. This observation is corroborated by Morsy et al. (2014), wherein a rat model of gentamicin-induced nephrotoxicity was used and the levels of serum creatinine, blood urea nitrogen, and urine albumin were significantly reduced after administration of sildenafil. In a similar way, Youssef et al. (2015) found that giving rats furosemide greatly decreased the amounts of serum creatinine and blood urea nitrogen after they had bilateral renal ischemia/reperfusion. All together, these results show that these agents, especially their nanoparticle forms, have the potential to help treat signs of kidney damage in ARF models.

Reactive oxygen species are critical mediators in ARF pathogenesis. These oxygen-based free radicals can cause lipid peroxidation in cell membranes, which leads to renal tubular necrosis (Irazabal and Torres 2020). As a result, lipid peroxidation emerged as a significant factor contributing to renal damage in our investigation. The therapeutic regimens involving sildenafil, furosemide, and their combined application, both in traditional and in NP formulations, ameliorated these detrimental markers. In particular, the nanoparticulate versions showed better results, which shows that our agents may have therapeutic value in managing ARF.

Our observations are similar to those of Ali et al. (2018), who found that giving sildenafil (2–5 mg/kg) greatly reduced MDA levels in a model of kidney disease caused by adenine. In a similar context, Helmy et al. (2014) documented that sildenafil therapy not only reduced MDA levels but also augmented nitrite/nitrate concentrations in male rats subjected to cisplatin-induced nephrotoxicity. Additionally, Khosravi et al. (2021) delineated that furosemide administration post-cisplatin exposure led to a decline in MDA and a surge in nitrite levels.

Neutrophil gelatinase–associated lipocalin is expressed in various organs. Under physiological conditions, its expression in the kidneys, trachea, and gastrointestinal tract is relatively subdued. But when there is an ischemic event, NGAL secretion quickly rises in the thick ascending limb of the renal tubules (Devarajan 2010). Tanase et al. (2019) have posited KIM-1 as a potential biomarker for renal injury, which could be useful in detecting and monitoring nephrotoxic agents. While KIM-1 remains undetectable in healthy kidneys, its expression surges in the context of renal dysfunction (Ichimura et al. 2004). Notably, some studies have highlighted that post-renal injury, KIM-1 mRNA levels exhibit a more pronounced elevation than any other gene (Wu et al. 2017).

In our investigation, we identified NGAL and KIM-1 as reliable markers for AKI. Our findings revealed that interventions with sildenafil, furosemide, and their combined regimen, in both traditional and NP formulations, led to significant reductions in NGAL and KIM-1 concentrations in the glycerol-induced AKI model. Impressively, the nanoparticulate versions of furosemide and their combined application demonstrated superior efficacy. Our therapeutic agents likely ameliorated glycerol-induced AKI by reducing NGAL and KIM-1 levels. In line with our findings, Hussein et al. (2022) saw lower levels of KIM-1 in a group of rats who had been exposed to gentamicin-induced nephrotoxicity after taking sildenafil. In the same way, Lahoud et al. (2015) found that pre-treatment with phosphodiesterase-5 inhibitors decreased the amount of KIM-1 that was excreted in the urine, but it did not change the levels of NGAL. In addition, Mose et al. (2019) found that giving healthy people furosemide stopped the rise in NGAL and slowed down the rise in urinary KIM-1 after a 3% saline infusion.

Acute renal failure studies in the past have shown that giving glycerol to rats causes glomeruli to break, bleeding, and vacuolization of medullary tubular cells, which was confirmed by microscopy (Singh et al. 2011). These observations are consistent with the results of our study on the histological changes seen in glycerol-induced ARF. Furthermore, our study shows that giving sildenafil, furosemide, or both together, in both standard and NP forms, can improve ARF. This is shown by the fact that the histological results were better in our study.

Damaged free radicals and inflammatory reactions are associated with ARF. Inflammatory cytokines are pivotal in the onset and progression of ARF. It is well known that patients with ARF have increased levels of numerous inflammatory cytokines, such as TNF-α and IL-1β (Simmons et al. 2004). Moreover, glycerol administration can notably increase the plasma levels of several inflammatory cytokines, including TNF-α and IL-1β (Amirshahrokhi 2021). According to Zhan et al. (2013), oxidative stress, which directly contributes to the development of ARF, can also trigger mitochondrial apoptosis and worsen renal dysfunction. Consequently, we analyzed the levels of the apoptotic protein caspase-3. Caspase-3, a key player in programmed cell death (apoptosis), has been found to be a marker of cell death in the kidneys and may play a role in glycerol-induced ARF. Furthermore, Al-Brakati et al. (2021) view the activation of caspase-3 as a crucial step in apoptosis, playing a significant role in the formation of apoptotic bodies and the loss of cell functionality.

Our study’s immunohistochemistry analysis, which aligns with the findings of Homsi et al. and Li et al., indicates a significant increase in the protein expression of IL-1β and caspase-3 in the renal cortex in cases of glycerol-induced ARF (Homsi et al. 2006; Li et al. 2019). This could help us understand how treatments with sildenafil, furosemide, or both of them together, in their regular and NP forms, can help with glycerol-induced ARF by lowering the levels of IL-1β and caspase-3 proteins. We observed a more noticeable improvement with the NP forms of sildenafil and furosemide.

The results of this study strongly suggest that sildenafil and furosemide, in both their standard and nanoparticle versions, have a significant positive impact on kidney protection in glycerol-induced ARF cases. Combining furosemide and sildenafil in their usual forms appears to enhance kidney function more than using these drugs separately. Moreover, the nanoparticle versions of these drugs showed superior performance compared to their standard counterparts, whether used individually or in combination. However, there are currently no clinically approved nanoparticles specifically designed to target the kidney for treatment or imaging purposes. Further studies are required to assess the efficacy of drugs loaded into nanoparticles in treating kidney diseases.

## Conclusion

The results indicate that the administration of sildenafil and furosemide, both individually and in combination, in both traditional and nanoparticulate formulations, enhances the amelioration of ARF in a glycerol-induced rat model. Notably, the combined nanoparticulate formulations exhibit pronounced efficacy. This observation underscores the potential therapeutic implications for ARF management.

## Limitation section

We should acknowledge the study’s limitations. Firstly, the study used the glycerol-induced acute renal failure model in rats, which may not fully replicate the complexity of human acute renal failure. Secondly, the study investigated both traditional and nanoparticle formulations of sildenafil and furosemide, but did not compare them to other potential drug delivery methods, implying that the effectiveness of these drugs may vary with different delivery methods. Thirdly, the study did not appear to investigate the long-term effects of the drug combinations on kidney function or overall health, leaving the long-term safety and efficacy of these treatments’ unknown. Fourth, the study appears to analyze the effects of drug combinations at a single time point after the induction of acute renal failure, which may not provide a complete picture of the disease’s progression and recovery. Lastly, this rat model did not account for factors such as age, sex, underlying health conditions, and genetic variability, which can influence the development and treatment of acute renal failure in humans.

## Data Availability

No datasets were generated or analysed during the current study.

## Abbreviations

AKI: Acute kidney injury
ARF: Acute renal failure
CS: Chitosan
cGMP: Cyclic guanosine monophosphate
GFR: Glomerular filtration rate
IL-1β: Interleukin-1 beta
KIM-1: Kidney injury molecule-1
MDA: Malondialdehyde
NPs: Nanoparticles
NGAL: Neutrophil gelatinase–associated lipocalin
NO: Nitric oxide
PDE5: Phosphodiesterase type 5
PLGA: Poly lactic-co-glycolic acid
TEM: Transmission electron microscope
